# Specialized Pro-Resolving Mediators Mitigate Cancer-Related Inflammation: Role of Tumor-Associated Macrophages and Therapeutic Opportunities

**DOI:** 10.3389/fimmu.2021.702785

**Published:** 2021-06-30

**Authors:** Margot Lavy, Vanessa Gauttier, Nicolas Poirier, Sophie Barillé-Nion, Christophe Blanquart

**Affiliations:** ^1^ OSE Immunotherapeutics, Nantes, France; ^2^ Université de Nantes, Inserm UMR 1232, CRCINA, Nantes, France

**Keywords:** inflammation, cancer, specialized pro-resolving mediators, macrophages, phagocytosis, tumor microenvironment

## Abstract

Inflammation is a fundamental physiological response orchestrated by innate immune cells to restore tissue homeostasis. Specialized pro-resolving mediators (SPMs) are involved in active resolution of inflammation but when inflammation is incomplete, chronic inflammation creates a favorable environment that fuels carcinogenesis and cancer progression. Conventional cancer therapy also strengthens cancer-related inflammation by inducing massive tumor cell death that activate surrounding immune-infiltrating cells such as tumor-associated macrophages (TAMs). Macrophages are key actors of both inflammation and its active resolution due to their plastic phenotype. In line with this high plasticity, macrophages can be hijacked by cancer cells to support tumor progression and immune escape, or therapy resistance. Impaired resolution of cancer-associated inflammation supported by TAMs may thus reinforces tumor progression. From this perspective, recent evidence suggests that stimulating macrophage’s pro-resolving functions using SPMs can promote inflammation resolution in cancer and improve anticancer treatments. Thus, TAMs’ re-education toward an antitumor phenotype by using SPMs opens a new line of attack in cancer treatment. Here, we review SPMs’ anticancer capacities with special attention regarding their effects on TAMs. We further discuss how this new therapeutic approach could be envisioned in cancer therapy.

## Introduction

Inflammation is now recognized as a hallmark of cancer (1) and several lines of evidence have highlighted the significance of chronic inflammation in fueling tumor progression and influencing tumor response to treatment in many cancers. Rudolf Virchow was the first to describe leukocytes within tumor in the 19^th^ century (2). A century later, Dvorak evidenced common features shared by inflammation and carcinogenesis proposing tumors as “wounds that do not heal” (3). This new inflammatory vision of tumor biology is now well supported by numerous studies that have highlighted the main contribution of chronic inflammation in cancer progression (4, 5). Inflammation is a defensive and protective reaction that aims to heal and repair damaged tissues. This physiological response is self-limited to prevent further detrimental over-reactions for tissue homeostasis. When inflammation is improperly regulated, deleterious signals may appear in inflamed tissues, supporting chronic inflammation setting up and fostering the development of many diseases, including autoimmune disorders, cardiovascular diseases or cancers. Resolution of inflammation is therefore a fundamental mechanism which allows damaged tissues to return to homeostasis in a process termed catabasis (6, 7). This active process mainly relies on a recently discovered array of molecules named specialized pro-resolving mediators (SPMs), including lipoxins, resolvins, protectins and maresins, which production is temporally and spatially regulated during acute inflammation (8, 9). SPMs mediate an effective inflammation resolution by transducing anti-inflammatory and pro-resolving signals that stop leukocytes recruitment in inflamed tissues and increase removal of apoptotic polymorphonuclear neutrophils (PMNs) (10). These events are crucial to break down further inflammatory signals and ensure restoration of tissue integrity. Among their target cells, SPMs have shown the ability to modulate macrophages’ biology by notably increasing their nonphlogistic (*i.e.* noninflammatory) phagocytosis of apoptotic PMNs. This phenomenon is named efferocytosis, from the Latin word ‘*effere*’ that translates to ‘to take to the grave’ (9, 11). Macrophages display tumor supporting roles and immunosuppressive functions that are exacerbated during carcinogenesis and tumor response to conventional anticancer treatments. SPMs may thus offer the opportunity to re-educate tumor-associated macrophages (TAMs) by promoting their pro-resolving activities and turning them toward an antitumoral phenotype.

Here, we summarize the recent findings on SPMs and cancer-focused macrophage biology as well as their connections. We further discuss the therapeutic opportunities to manipulate tumor and inflammation dialogue during cancer progression or therapeutic pressure especially through TAMs re-education using SPM-based therapies to improve anticancer treatments.

## SPMs Mediate Resolution of Inflammation

### SPMs’ Roles in Inflammation Resolution

**Figure 1:**
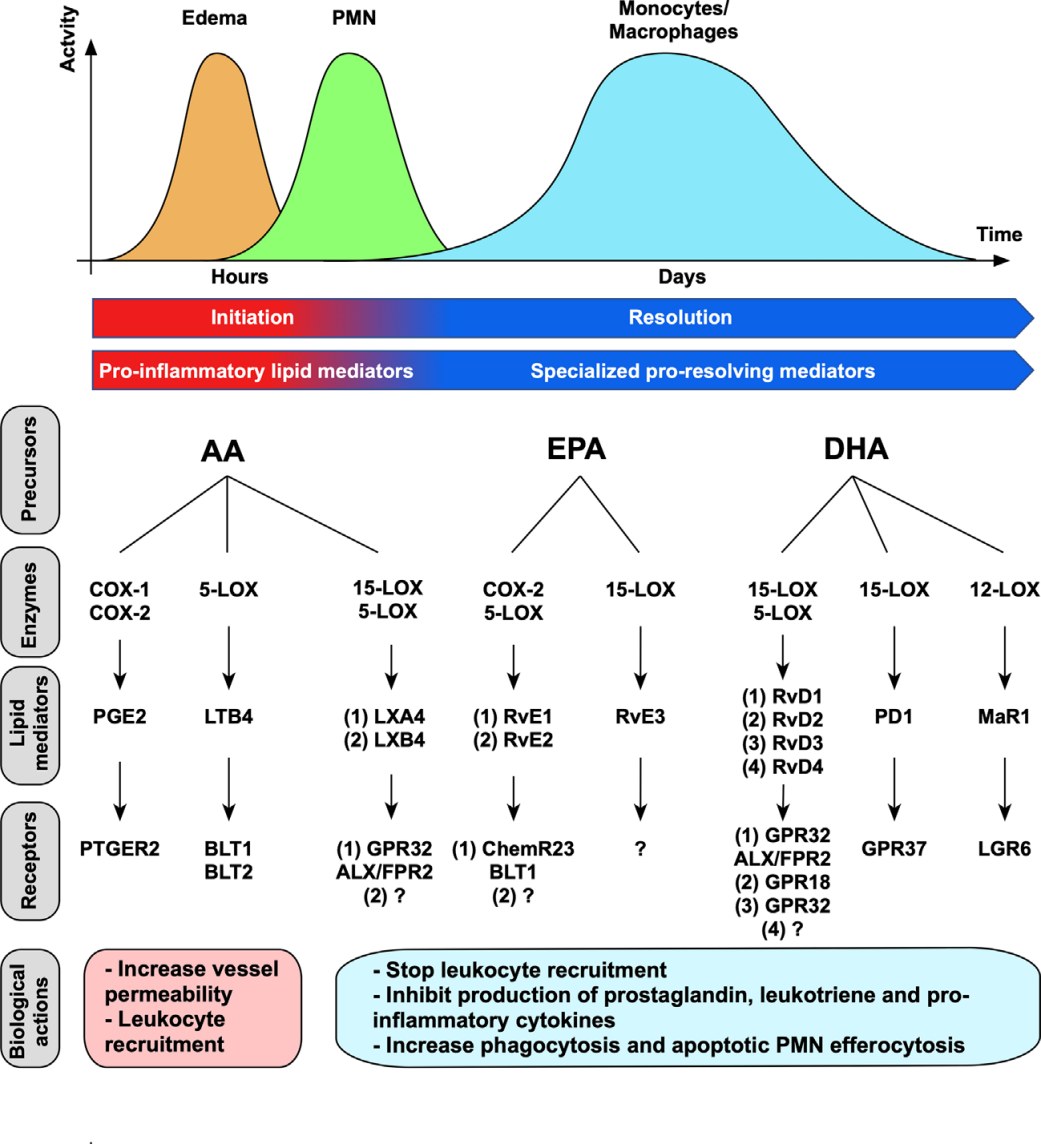
Lipid mediators’ time-course, synthesis and biological actions. Lipid mediators are produced sequentially through action of specific enzymes. Pro-inflammatory lipid mediators (prostaglandins and leukotrienes) are first produced during inflammation initiation. During inflammation resolution, a switch in lipid mediator synthesis occurred and promote SPMs’ synthesis. Each SPM execute its functions by binding to a specific receptor. The receptors for LXB4, RvE2, RvE3, RvD4 have not been characterized yet (14–17).

Acute inflammation is a finely regulated physiological process initiated within minutes to hours in response to tissue injury or infection, where cellular and chemical mediators are operating. Pro-inflammatory lipid mediators including prostaglandins (PGs) and leukotrienes (LTs) are first secreted during the initiation step. Prostaglandin E2 (PGE2) contributes to the dilatation and increase in permeability of vascular vessels, and leukotriene B4 (LTB4) to leukocyte chemotaxis, both mechanisms are necessary for inflammation amplification (7, 12, 13). Increased blood flow and vessels permeability induce fluid, proteins and leukocytes to migrate to the inflammatory site, resulting in swelling (edema) (**Figure 1**). The first cellular effector recruited in damaged site are neutrophils (9) that kill pathogens due to their microbicidal activities and ensure debris removal by phagocytosis. Monocytes are subsequently recruited by chemotactic factors and differentiate locally into macrophages that clear cellular debris and apoptotic PMNs. Importantly, during inflammation progression, a switch in lipid mediator synthesis from pro-inflammatory (PGs and LTs) to pro-resolving mediators (SPMs) occurs in order to trigger inflammation resolution. Lipoxins’ (LXs) production first occurs both locally and systemically to stop leukocyte recruitment and is then completed by a production of other SPMs to exert their pro-resolving effects. These effects include the cessation of neutrophils’ recruitment and the increase of apoptotic PMNs removal by macrophages (7, 18–20). Efferocytosis is an important physiological process where phagocytes engulf apoptotic cells before they become necrotic and release danger molecules, thus preventing further exacerbated inflammatory signals (21). In addition, apoptotic PMNs’ efferocytosis by macrophages induces a reparative phenotype by triggering intracellular signaling that further contribute to inflammation resolution through production of TGF-β and VEGF, that are necessary for tissue repair (7). Therefore, the switch in bioactive lipid production from PGs and LTs to SPMs is fundamental to prevent inflammation exacerbation. This mechanism is required for complete resolution and return to tissue homeostasis (9).

### SPMs’ Biosynthesis and Interactive Network

SPMs are a set of recently identified lipid mediators that act locally as endogenous agonists to stimulate the resolution of inflammation. Four subclasses of SPMs have been identified so far: lipoxins, resolvins (Rv), protectins and maresins (MaR) (10, 22). The discovery of these new bioactive lipids changed the comprehensive view of inflammation resolution as it was initially considered a passive process where inflammatory cells and chemical mediators were locally diluted (23). The emergence of the SPM family as omega-3 polyunsaturated fatty-acid (PUFA) derived bioactive lipids revealed their crucial role in actively “turning off” the inflammatory response by stopping neutrophils infiltration and enhancing their efferocytosis by macrophages (9). This active catabatic process is temporally and spatially regulated to sustain tissue homeostasis (8). However, evidence of chronic inflammation in different clinical contexts that initiates or fuels pathogenesis, reflects a defect in the temporal regulation of inflammation resolution. Under these conditions, SPMs may represent actionable targets to “switch on” resolving mechanisms and improve treatment outcome. Recent studies have highlighted the potential antitumor roles of SPMs in carcinogenesis and cancer progression including tumor response to treatment. Therefore, a comprehensive understanding of their biology is required to manipulate them for therapeutic purposes.

Lipid mediators include both pro-inflammatory (PGs and LTs) as well as anti-inflammatory and pro-resolving mediators (SPMs). They are generated through the action of the specific enzymes cyclooxygenases (COXs) and lipoxygenases (LOXs) from PUFA, including arachidonic acid (AA), eicosapentaenoic acid (EPA) and decosahexaenoic acid (DHA) (6) (**Figure 1**). Among COXs, COX-1 is constitutively expressed in several tissues and is responsible for basal level of PGs that are necessary for the maintenance of homeostasis, whereas COX-2 expression is triggered under inflammatory or tumorigenic signals (24). LOX enzymes present cell-specificity as 5-LOX is expressed in both myeloid (DC, monocytes and macrophages) and lymphoid (T- and B-cells) cells, 12-LOX in platelets and macrophages, and 15-LOX in vascular cells (12). Importantly, SPMs’ production involves sequential enzymatic reactions that occur during cell-cell interactions. Arachidonic acid is metabolized to lipoxins through action of 15-LOX and 5-LOX during interactions between inflammatory (neutrophils or macrophages) and vascular cells (endothelial cells or platelets) (8). Resolvins are either named E-series or D-series resolvins that are respectively derived from EPA or DHA (14). Among E-series resolvins, RvE1 and RvE2 are produced by sequential actions of cytochrome P450 or COX-2 acetylated by aspirin in endothelial cells, and 5-LOX in human PMNs (25). D-series resolvins are produced through successive actions of 15-LOX and 5-LOX. Importantly, systemic administration of aspirin promotes acetylation of COX-2 which changes its enzymatic activity (*i.e.* stopping prostaglandins’ production) to generate epimeric forms of SPMs termed aspirin-triggered SPM (AT-SPM) (25). Of note, AT-LXA4 and AT-RvD1-2-3-4 have been described to date (14). Protectins are derived from DHA and produced through action of 15-LOX in leukocytes (14). Maresin is the latest SPM family discovered and is derived from DHA, and MaR1 is synthesized *via* 12-LOX by macrophages (26). SPMs’ synthesis is finely orchestrated to guarantee their right sequence of actions locally to resolve acute protective inflammation.

Of importance, SPMs are short-lived molecules that are further metabolized and inactivated by various enzymatic pathways involving β-oxidation, ω-oxidation, dehydrogenation and reduction (27). They act through specific G-protein-coupled receptors (GPCRs) (23) in a picogram to nanogram dose range (**Figure 1**). GPCRs represent the largest family of cell surface receptors, counting around 800 members (28), and a complex network of interactions has been already described between SPMs and GPCRs. In fact, two SPMs can bind to the same GPCR. For example, LXA4 and RvD1 both bind GPR32 and ALX/FPR2 expressed on PMNs and monocytes (6, 29–32). In addition, one SPM can bind to two GPCRs with different affinity. For instance, RvE1 binds to ChemR23 on myeloid cells, including macrophages and dendritic cells, and acts as a partial agonist for BLT1 expressed on PMNs to counteract LTB4’s pro-inflammatory actions (8, 33). GPCRs can also interact with ligands of different nature, as evidenced for ChemR23, that binds both RvE1 and the chemoattractant protein chemerin, consequently strengthening the complexity of this network. The deep understanding of the interconnected systems linking SPMs to their specific GPCRs still offers many approaches for targeting these inflammation controlling networks.

### SPMs and Tumor: Clinical Correlations

Importantly, unresolved inflammatory process leads to chronic inflammation with significant pathogenic consequences such as organ damages, auto-immune diseases or cancer initiation and progression (6, 32). Deficiency in SPMs’ synthesis or biological activities participates to chronic inflammation establishment that sets the stage for tissue damage. For example, LXA4 deficiency is associated with cystic fibrosis and severe asthma (34, 35). Regarding cancers, overexpression of COX-2, 5-LOX and high levels of PG- and LT-derived metabolites that have important roles in tumor progression have been reported in many tumors (36, 37). Contrarily, to date, few studies have investigated the potential relationship between SPM levels and cancer. Decreased levels of LXA4 induced by 15-LOX down-regulation in serum and tumor from patients with colorectal cancer (38, 39) have been reported, suggesting the anti-tumorigenic potential of 15-LOX-derived metabolites in colon carcinogenesis (38). In another study, lower serum levels of RvD1 were found in patients with colon cancer compared to healthy volunteers and were reversely correlated with advanced cancer stage (40). These serum levels were further correlated with better survival in patients with endometrial cancer, suggesting that RvD1 could be a predictive biomarker of high tumor blood flow (41). Altogether, these clinical data demonstrated that impaired SPM activity coincides with promotion of carcinogenesis. Of note, the non-steroidal anti-inflammatory drug (NSAID) aspirin has shown protective effects in colorectal cancer and reduced the risk of developing other cancers such as lung and breast cancers (42, 43). Importantly, anticancer effects of aspirin were in part mediated through irreversible COX-2 acetylation and the generation of AT-SPM (44). These data emphasize the therapeutic potential of preventing inflammation in cancer, especially by using SPMs or their mimetics, as developed in the fourth part of this review.

## Macrophages Are Guardians of Tissue Homeostasis and Key Regulators of Pro-Tumor Inflammation

### Diversity of Macrophages Phenotypes and Functions

Macrophages are part of innate immune cells that possess important functions in organ development and tissue homeostasis. Macrophages can refer to either tissue-resident or monocytes-derived macrophages, that have different functions in tissues. Tissue-resident macrophages mainly originate from the embryonic yolk sac and fetal liver progenitors (45). These macrophages activate a unique transcriptional program dependent on their tissue of residence, as illustrated by specialized liver Kuppfer cells, brain microglia, lung alveolar macrophages or bone osteoclasts (46, 47). They maintain their pool by local homeostatic proliferation (48) and can be enriched locally from peripheral monocytes recruitment upon inflammatory signals. In contrast, monocyte-derived macrophages are continuously produced in bone marrow during hematopoiesis, and are recruited from blood circulation during inflammatory process. Both types of macrophages differentially exert their functions in damaged tissues. In sterile microlesions, tissue-resident macrophages prevented initiation of inflammation by sequestrating cell debris. When tissue-resident macrophages could not control the surging inflammatory signals (macrolesions or successive microlesions), PMNs’ swarming triggered monocytes recruitment that in turn, engaged resolving and reparative programs (49). Distinctively from tissue-resident macrophages that have a major first-line homeostatic function, monocytes-derived macrophages are involved in pro-resolving mechanisms.

To ensure tissue homeostasis, macrophages have a myriad of functions, including host defense against pathogens or damage and regulation of immune responses to ensure tissue repair in these contexts. Macrophages are part of classical antigen-presenting cells (APCs) and mediate cellular immune responses through the processing and presentation of antigens to lymphocytes (50). In addition, macrophages are professional phagocytes that engulf either foreign particles (microbes) or altered self-particles or cells (dying or dead cells) (51, 52). They can quickly produce a large array of cytokines and chemokines that orchestrate local acute inflammatory process (52, 53).

Both diversity and plasticity define macrophages since they can acquire different phenotypes and functions depending on the stimuli present in the surrounding environment. Such properties are supported by plasticity of the epigenetic modifications that can rapidly modify macrophages’ identity and destiny depending on external stimuli (54, 55). Those modifications includes DNA methylation, histone modifications and non-coding RNA (56). Of note, microRNA generated in response to various environmental stimuli have been reported to modulate macrophages’ final differentiation (54).

**Figure 2:**
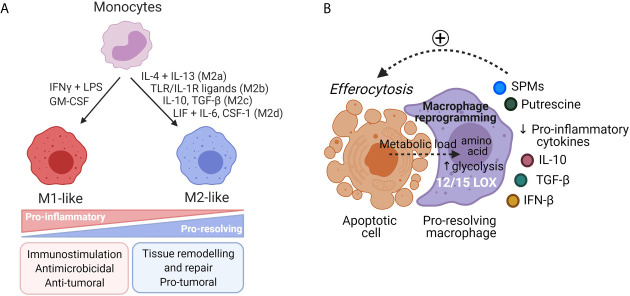
Diversity of macrophages’ phenotypes. **(A)** Monocytes can be differentiated *in vitro* either in pro-inflammatory M1-like macrophages or in anti-inflammatory M2-like macrophages upon various external stimuli. M1-like macrophages are involved in microbicidal and tumoricidal activities. M2-like macrophages have pro-resolving functions and are involved in tissue repair. In cancer settings, TAM, associated to a M2-like phenotype, support tumor progression and mitigate tumor response to anticancer treatments through production of proteases, angiogenic and growth factors. **(B)** During efferocytosis of apoptotic cells, macrophages undergo a reprogramming toward a pro-resolving phenotype. Macrophages receive an important metabolic load from these engulfed cells that induce metabolic modifications such as activation of the putrescine pathway or increased glycolysis that supports actin polymerization and cell clearance. Pro-resolving macrophages express 12/15-LOX and turn off production of pro-inflammatory cytokines for production of IL-10, TGF-β and IFN-β that participate in inflammation resolution.

Macrophages are generally classified as pro-inflammatory M1-like or anti-inflammatory M2-like macrophages (47) (**Figure 2A**). M1-like macrophages exert pro-inflammatory properties and are involved in microbicidal and tumoricidal activities. They can be generated *in vitro* in response to IFN-γ, GM-CSF or Toll-like receptors (TLR) agonists. They are characterized by secretion of high level of IL-12 and low levels of IL-10, production of nitric oxide (NO) and reactive oxygen species (ROS), secretion of pro-inflammatory cytokines including TNF-α and IL-1β (57, 58). In contrast to M1-like macrophages, in physiological contexts, M2-like macrophages are involved in inflammation resolution and wound healing. Mantovani et al. proposed a classification for M2-like phenotypes depending on stimuli used for monocyte-derived macrophages differentiation. Monocytes stimulation by: (1) IL-4 and IL-13 leads to M2a phenotype involved in tissue repair and killing of extracellular pathogens (59, 60); (2) Immune complexes or TLR or IL-1R ligands to M2b phenotype involved in immune regulation (61); (3) Glucocorticoids or IL-10 to M2c phenotype involved in efferocytosis of apoptotic PMNs and resolution of inflammation (62); and (4) CSF-1 or adenosine or IL-6 plus LIF to M2d phenotype involved in angiogenesis and immunosuppression. M2d macrophages reflect *in vivo* TAM phenotype with pro-tumorigenic properties (63).

Regarding phagocytic properties, M2-like macrophages can phagocyte more microbial particles and cancer cells than M1-like macrophages. Of interest, macrophage expression of specific surface markers can reflect their phagocytic capability (CD14, CD206 and CD163) or even their phagocytic capacity (i.e. amount of particles; CD209) as evidenced by a mass cytometry-based phagocytosis assay (64).

Importantly, M1-like and M2-like monocyte-derived macrophages produce distinct lipid mediator profiles. For example, upon bacterial stimulation, human M1-like macrophages display increased production of 5-LOX-derived LT including LTB4 and COX-2-derived PGs including PGE2, which production was 20 times higher in M1-like compared to M2-like macrophages. In contrast, M2-like macrophages exhibit increased 15-LOX expression resulting in SPM production that represent 50% of their total lipid mediators synthesis (65–67).

Generally, M1-like macrophages are associated with pro-inflammatory, antiviral, antibacterial, antitumor phenotypes and with production of pro-inflammatory lipid mediators (PGs and LTs). For their part, M2-like macrophages are defined by anti-inflammatory and reparative phenotypes related to their production of pro-resolving SPMs, but are also associated to a protumoral phenotype in cancers. However, this M1/M2 *in vitro* polarization model has shown its limitation as it does not authentically reflect macrophages’ polarization occurring in tissues. It rather is a continuum between M1-like and M2-like phenotypes depending on cytokines present in the surrounding environment (68) and the origin of macrophages (69). This classification is still useful to study macrophages’ biology *in vitro* but for greater clarity, Murray et al. suggested to adopt a specific nomenclature that specifies the stimuli used for monocyte activation [i.e. M(IL-4) for macrophages activated *in vitro* by IL-4] (70).

### Pro-Resolving Functions of Macrophages

Removal of apoptotic cells by macrophage-dependent efferocytosis is a key step to achieve complete local inflammation resolution and return to tissue homeostasis. Efferocytosis of apoptotic PMN increased macrophages’ SPMs biosynthesis (RvE1, PD1, LXA4, MaR1) (71) that in turn acts as a positive feedback loop, as evidenced by LXA4, that enhanced nonphlogistic PMN engulfment by macrophages (65, 72). Moreover, SPMs production by macrophages is enhanced through the release of microparticles that contain metabolic precursors, derived from dying neutrophils at the inflammatory site (65). Both efferocytosis and microparticles derived from apoptotic cells trigger macrophages’ reprogramming from a pro-inflammatory to a pro-resolving hybrid phenotype between M1-like and M2-like macrophages (**Figure 2B**) (65, 73). Pro-resolving macrophages undergo continuous efferocytosis of apoptotic cells to prevent their evolution toward necrosis until complete inflammation resolution. This process is upregulated by metabolites loaded from apoptotic cells during the first round of efferocytosis and the putrescine pathway (74). Apoptotic cells ingestion induces a deep metabolic reprogramming in efferocytic macrophages (75) that blocks the production of pro-inflammatory cytokines. Moreover, this process triggers the secretion of factors including IL-10, TGF-β, CCL-5 and protein S that promotes the clearance of apoptotic cells, prevents autoimmunity, and further supports macrophages’ reprogramming (76–79).

So far, pro-resolving macrophages’ phenotypes and functions have been mainly described in murine models of acute inflammatory diseases. Macrophages isolated from resolving acute peritonitis produced high levels of anti-inflammatory IL-10 and expressed M1-like markers such as iNOS and COX-2. They also expressed high level of cAMP that triggers COX-2 dependent lymphocyte repopulation necessary to restore homeostasis post inflammation, and prevent further reinfection (80). Importantly, transcriptomic analyses revealed that murine resolution-phase macrophages possess a unique phenotype that cannot fit the conventional M1/M2 classification, conferring them a hybrid phenotype with shared characteristics of M1-like and M2-like macrophages (81). Resolution-phase macrophages are enriched with molecules involved in antigen presentation and T-/B-cell chemotaxis, they produce SPMs, and they are involved in phagocytosis of apoptotic cells (82). Of particular interest, a specific population of “satiated” macrophages have been described among resolution-phase macrophages in zymosan-A induced peritonitis. This population is characterized by downregulation of CD11b expression induced by efferocytosis, production of high levels of anti-inflammatory and pro-resolving cytokines, including TGF-β, and high expression of 12/15-LOX. CD11b^low^ macrophages were termed “satiated” as they have ingested many apoptotic PMNs but are no longer capable of phagocytosis, contrarily to CD11b^hi^ macrophages from which they derive. CD11b^low^ macrophages then migrate out of the inflammatory site (83). These data were reinforced by transcriptomic analyses that demonstrated a down regulation of gene clusters involved in phagocytosis and cytoskeleton configuration, and increased expression of gene clusters involved in myeloid cell migration (84). Moreover, non-phagocytic macrophages produce IFN-β that actively increases PMNs’ apoptosis and removal by efferocytosis and participates in phagocytotic macrophage reprogramming (85). These results obtained in inflammatory murine models have clarified the vision of pro-resolving macrophages and the importance of their phenotypic and functional switch that finely regulates inflammation resolution. Of major interest, restoring pro-resolving functions of macrophages using SPMs in murine models of cancer deeply impaired tumor progression (86, 87). However, since mouse and human macrophages exhibit different gene signatures and metabolisms, efforts must now be concentrated on unraveling the profile of human pro-resolving macrophages (88). It would also demonstrate that promoting resolution of inflammation represent a promising strategy for the treatment of inflammatory diseases including cancers.

### Macrophages Have Pro- But Also Anti-Tumor Activities in Cancer

Many observations have revealed tight links between cancers and inflammation (4). Inflammation may initially arise from cancer-associated exogenous pathogens (5) or from tumor cells secreting various pro-inflammatory factors (2, 89). When persisting, inflammation feeds carcinogenesis and cancer progression. Importantly, anticancer treatments including chemotherapies and radiotherapies can also trigger an inflammatory response at the tumor site resulting in a protumoral environment that supplies tumor progression and immunosuppression (86, 90).

Macrophages infiltrated in tumors are defined as tumor-associated macrophages (TAMs). TAMs first arise from tissue-resident macrophages (91) but various tumor-produced chemokines (CCL-2, CCL-5) and cytokines (CSF-1) also recruit circulating monocytes and further promote their differentiation into TAMs (92–94).

Importantly, macrophages are the major immune infiltrating component of tumor microenvironment (TME) in many solid tumors, representing up to 36% of total immune cells in breast tumors (95–97). TAMs high density coincides with a poor prognosis in many cancers (bladder, prostrate, ovarian and breast cancer) excepted for colorectal cancer (98). Recently, new technologies based on deconvolution of gene expression profiles or single cell analysis by RNA or mass-cytometry have gained great interest in studying TAMs (99, 100). Using these approaches, TAMs have been classified in clusters, characterized by differential expression levels of M2 markers such as CD163, MARCO and CCL-18 (101–103). Infiltration of M2-like TAMs has been associated with lower overall survival in non-small cell lung cancer (NSCLC) (104), lung adenocarcinoma (105) and gastric cancer (106). Importantly, these recent data demonstrated that TAMs expressed both M1 and M2 gene signatures (101, 102, 107, 108) and that presence of M1-like TAM was associated with increased survival in NSCLC (109) and in advanced ovarian cancers (110). Interestingly, upregulation of genomic signatures associated with immune cell activation and antigen presentation or phagocytosis, immune responses and blood vessels formation has been revealed in TAMs, respectively isolated from breast and endometrial cancer patients, compared with tissues-resident macrophages (111). Of note, a specific TAM subset associated with a phagocytic pattern has been reported in lung, colorectal, ovarian and breast cancer (108).

Those data have weakened our vision of the binary macrophage polarization model since TAMs may represent a specific “cancer-associated” spectrum of alternative polarization states between M1-like and M2-like macrophages. These data have also provided a new perception of TAMs’ complexity as they demonstrated that TAMs both express pro- and anti-tumor markers (112).

**Figure 3:**
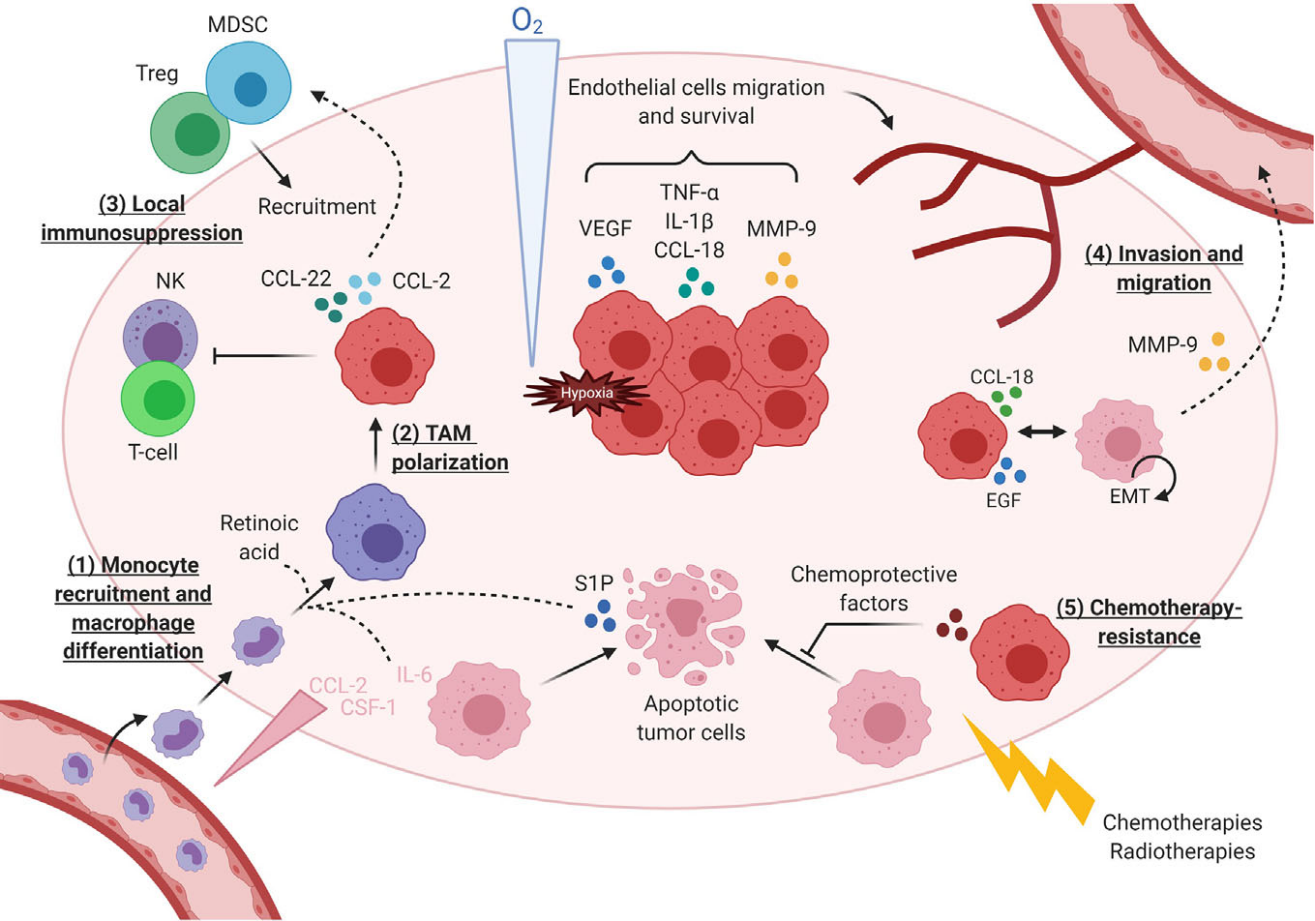
TAMs execute diverse activities during tumor initiation and its immune escape. (1) Macrophages are first recruited in tumor under the action of chemokines secreted by tumor cells. Some factors such as retinoic acid (113) and IL-6 (114) favor monocytes differentiation toward macrophages instead of dendritic cells. (2) Macrophages become TAMs with immunosuppressive activity, in particular due to sphingosine 1 phosphatase (S1P) released by dying tumor cells. (3) TAMs support local immunosuppression by: secreting immunosuppressive cytokines, inhibiting effector T-cells (115) and NK cells (116), recruiting Tregs though CCL22-CCR4 axis or MDSC through CCL2-CCR2. (4) Under hypoxia, macrophages produce various cytokines, including CCL-18 (117) triggered by tumor cells at the tumor invasive front, and proteases that induce EMT in tumor cells and favor their invasion and migration. Additional paracrine loops between tumor cells and TAMs, as described for EGF and CSF-1, amplify TAM-dependent cancer cell motility (94, 118). (5) Finally, TAMs contribute to anticancer treatment resistance by secreting factors protecting tumor cells from death.

Many studies have reported that TAMs display strong tumor supportive activities during both primary tumor development and its metastatic progression (**Figure 3**). TAMs produce prosurvival factors that protect tumor cells from apoptosis (PGE2, IL-10, TGF-β and IL-6) (119–121), including from chemotherapies-induced cell death (122). TAMs accumulate particularly in hypoxic areas (123, 124) where they secrete proangiogenic factors and proteases (VEGF, β-FGF, TP, TNFα, CXCL18, uPA, CCL18) that support endothelial cells’ survival and migration for the development of a high-density vascular network (95, 124). Among proteases, TAMs produce MMP-9 that induce extracellular matrix remodeling further supporting tumor cell invasion (125, 126). TAMs also operate at distant sites during metastatic processes as observed in murine breast tumor cells that, when reaching lung capillaries, secrete CSF-1 to recruit new macrophages that in turn, help them to invade the lung parenchyma (127). In addition, TAMs actively participate in promoting an immunosuppressive TME through expression of immune checkpoints (PD-1 and B7-H4), production of immunosuppressive cytokines (IL-10 and TGF- β) (128), recruitment of immunosuppressive cells (Tregs and MDSC) (116, 126), and inhibition of effector immune cells (NK and T-cell) (115, 116). Importantly, chemotherapies often induce macrophages’ recruitment in tumors, as seen by increased CD68+ macrophages infiltration in matched breast tumor samples before and after neoadjuvant chemotherapy (90, 122). Recruited macrophages presented a reinforced M2-like phenotype (90, 129, 130). Moreover, macrophage abundance in treated tumors was correlated with a low cytotoxic T cell infiltrate, suggesting that chemo-recruited TAMs increase immunosuppression by limiting T-cell responses to anticancer treatment (90).

Despite their tumor-supporting roles, macrophages constitute a high proportion of immune effectors in the majority of liquid and solid tumors, particularly in the so called “cold” tumors, and can be manipulated to eradicate tumors. Macrophages can exert direct (cell-contact, independent of phagocytosis) or indirect (soluble effectors) cytotoxicity toward tumor cells, at least *in vitro (* 94, 131). They can recognize tumor cell through expression of various receptors (lectins, phosphatidylserine, integrins) (132) and produce when activated, various cytotoxic molecules [NO (94)] and cytokines [TNF-α (133)] that trigger tumor cells apoptosis and phagocytosis. Macrophages within tumors can be activated by tumor-associated antigens that they can further process and present *via* their MHC-II to CD4+ helper T cells and CD8+ cytotoxic T cells (132), to promote effective antitumor adaptive immune responses. Loss of efficient antitumor functions of macrophages due to their hijacking by cancer cells is critical for the development of antitumor immune responses. For example, increased expression of CD47 by tumor cells triggers an inhibitory signaling in macrophages through the immune checkpoint SIRPα, that limits macrophage-dependent phagocytosis (134) and lymphocyte chemotaxis (135). Restoring and/or stimulating antitumor functions of macrophages within tumors may counterbalance their tumor-supporting roles and help to promote an efficient immune antitumor response.

### TAMs Are Preferentially Associated With a Pro-Inflammatory Lipid Mediator Profile in Cancer Context

Because of highly specialized approaches to detect and quantify lipidic mediators in biological specimens, TAM-related lipid mediators’ profile has not been extensively studied. However, metabolic enzymes and receptors involved in activities of these mediators can be appraised using transcriptomic analyses. As such, TAMs isolated from ascites of patients with ovarian cancer revealed that TAMs expressed *PTGS2* (coding for COX-2), PGE2 and LTB4 receptors, contrarily to tumor cells (136). These results suggest that TAMs could be associated with a pro-inflammatory lipid mediator profile based on PGs and LTs, that supports their pro-tumorigenic functions.

Of importance, cancer cells indirect interactions with TAM-like *in vitro* alter the lipid mediator profile produced by cancer cells toward the synthesis of pro-inflammatory PGs and LTs, with pro-tumorigenic properties (36, 137). Among PGs, PGE2 largely contributes to tumor progression and promotes an immunosuppressive TME, particularly in macrophages (37, 138). Reversely, cancer cells also affect macrophages’ lipid mediator profile through induction of COX-2 expression and release of sphingosine 1-phosphate (S1P) when dying. COX-2-derived PGE2 from both TAMs and cancer cells reinforce macrophages’ immunosuppressive phenotype and inhibit their phagocytic capability (139). Moreover, TAM-like recognition of apoptotic cancer cells induced reduction of 5-LOX expression in TAMs in breast tumor spheroid models. This result is in line with lower 5-LOX expression in macrophages from human breast invasive tumors compared with macrophages from normal breast tissues (137). TAMs with reduced 5-LOX expression have reduced capacity to recruit effector T cells, suggesting an antitumor role for 5-LOX (137). These data demonstrated that cancer cells set up an inflammatory environment supported by the generation of pro-inflammatory and tumor-supporting bioactive lipids. Importantly, it has been reported that TAM indirect interactions with lung cancer cells induced SPM production (RvE3, RvD2, RvD5) (66), but to a lower extent than PGs and LTs. To strengthen the switch of bioactive lipid mediators occurring during inflammation resolution, the promotion of SPMs synthesis within tumors would reduce inflammation and restore antitumor functions of macrophages in the TME. Indeed, as developed below, SPMs demonstrated the capacity to induce anti-inflammatory and antitumor responses in cancer context. Further studies are needed to fully characterize the expression of SPMs and their specific receptors in TAMs to better understand the implications of these pro-resolving molecules in cancers.

## Targeting TAMs Using SPMs as an Anticancer Strategy

Since inflammation fuels cancer progression, use of anti-inflammatory drugs such as NSAID was first envisioned to interfere with this deleterious process (140). However, new insights in inflammation biology suggest that promoting its active resolution rather than achieving its complete inhibition may help to foster an effective antitumoral immune response, in particular during anticancer treatments (89). In this regard, recent studies demonstrated that SPMs exerted intrinsic antitumor activities mainly through macrophages and greatly improve tumor response to chemotherapies in various murine cancer models (86). This innovative concept strengthened by the fundamental role of macrophages in both inflammation resolution and cancer progression, has gained great interest in cancer treatment (141). Macrophages depletion and/or recruitment inhibition using specific monoclonal antibodies, such as anti-CSF1R, were first investigated to breakdown their growth-supportive roles. However, important toxicity have been reported and linked to a depletion of all macrophages in the body (142). Instead of their therapeutic eradication, repolarization of TAMs toward an antitumoral phenotype based on high phenotypic plasticity opens new possibilities in cancer treatment (143). From this perspective, recent approaches are being evaluated: epigenetic reprogramming of M2-like TAMs into M1-like TAMs (144); increasing phagocytic activity by CD47/SIRPα blockade (145, 146); engineering Chimeric Antigen Receptors for Phagocytosis (CAR-P) to increase tumor cell killing (147, 148). Recent reports also illustrate that macrophages can be involved in immunotherapy resistance mechanisms such as T-cell exclusion by impeding CD8 T-cells from reaching tumor cells (149). Moreover, relieving the SIRPα break on macrophages promotes intra-tumoral chemokine secretion, T-cell migration in tumor bed and improves T-cell immunotherapy responses (135).

### SPMs Shape Macrophages Functions

In view of their crucial role in inflammation resolution, early studies using SPMs were first conducted in inflammatory contexts and aimed to understand SPMs’ pro-resolving effects on macrophages. Of note, some effects were mediated by SPM mimetics including SPM analogs and receptor agonists (named in bold in **Table 1**) that will be further developed in part 4. Even though experimental conditions vary between studies (*i.e.* type of macrophages, SPM concentration and time of incubation) they all demonstrated that SPMs increased macrophages’ phagocytosis of microbicidal particles and efferocytosis of apoptotic PMNs (**Table 1**). Those effects were accompanied by decreased macrophages’ secretion of pro-inflammatory cytokines (153, 156, 159) mediated in part by reducing the nuclear factor-κB (NF-κB) activation (33, 156, 159, 163), a transcription factor involved in the development and maintenance of chronic inflammation (164). SPMs such as RvD1 and RvD2 can also induce macrophages’ secretion of anti-inflammatory cytokines including IL-10 and TGF-β (156, 160), two cytokines involved in resolving and reparative mechanisms. In addition, LXA4 exerted death protective effects on macrophages (150), allowing them to ensure complete functions during inflammation resolution. These results underline a SPM-based phenotypic switch in macrophages toward an intermediate phenotype between M1-like and M2-like (155, 157, 159) with pro-resolving functions.

**Table 1:** SPMs’ *in vitro* effects on human macrophages in inflammatory contexts.

Macrophages	SPMs	Effects	References
Human monocyte-derived macrophages (M1-like, M2-like)	LXA4 (250nM)	Protect macrophages from apoptosis	(150)
	LXA4 (0.1-10nM)	Increase phagocytosis of zymosan particles	(151)
Human monocytes	LXA4 **(ATL-1)** (1-100µM)	Inhibit monocytes’ apoptosis	(152)
Primary human macrophages	LXA4 **(ATL-1)** (100µM)	Increase phagocytosis Decrease secretion of pro-inflammatory cytokines	(153)
Human monocyte-derived macrophages (M1-like)	RvD1 (0.1-1nM)	Increase phagocytosis of microbial particles and apoptotic PMN	(154)
	RvD1 (0.1-10nM)	Increase phagocytosis of zymosan particles	(151)
	RvD1 (10nM)	Switch M1-like to M2-like macrophages	(155)
Human alveolar macrophages	RvD1 and RvD2 (100 nM)	Increase phagocytosis of microbial particles (E. coli) Decrease secretion of pro-inflammatory cytokines	(156)
Primary human macrophages	RvD1 (10nM)	Polarize resting primary macrophages and repolarize M1-like macrophages to a pro-resolving phenotype	(157)
M1​-like macrophages	RvD5​ (10nM)	Increase phagocytosis of microbial particles	(67)
Human monocyte-derived macrophages (M1-like)	RvE1 (10nM)	Increase phagocytosis of microbial particles	(158)
Primary human macrophages	RvE1 (10nM)	Induce a pro-resolving phenotype	(159)
Human monocyte derived macrophages (GM-CSF)	RvE2 (1-10nM)	Increase phagocytosis of zymosan particles	(160)
Human macrophages (M2-like)	MaR1​ (1 nM)	Increase efferocytosis of apoptotic PMN	(161) ​
Human monocyte-derived macrophages (M1-like, M2-like)	MaR1​ (10pM-10nM)	Increase phagocytosis (E. coli, zymosan) and efferocytosis of apoptotic PMN	(19)
Human monocyte-derived macrophages (M1-like)	MaR1 (10 nM)	Switch M1-like to M2-like macrophages	(155)
Human macrophages	PD-1 **(22-F-PD1)** (0.001-10nM)	Increase macrophages’ efferocytosis of apoptotic PMN	(162)

Bold, SPM analogs or receptor agonists.

Importantly, as inflammation is an integrated part of cancer biology, SPMs’ effects on human macrophages have started to be evaluated in cancer contexts (**Table 2**). To better mimic macrophages’ polarization within tumors, biological fluids from cancer patients or conditioned media from tumor cells, are now used for *in vitro* monocytes differentiation into TAM-like macrophages (63, 165, 168). In such settings, SPMs including LXA4, RvD1 and RvD2 suppressed TAM-like phenotype, suggesting that SPMs can modulate macrophages’ polarization in cancer context (165, 167). Interestingly, SPMs’ functions have been tested in *in vitro* models of chemotherapy-mimicking inflammation (86). Incubation of monocyte-derived macrophages with chemotherapies-induced tumor cell debris in presence of RvD1, RvD2, or RvE1 increased their efferocytotic activity and reduced their secretion of pro-inflammatory cytokines (86). Moreover, LXA4’s analog decreased TAM-like’s production of IL-10, a cytokine associated with immunosuppressive properties in TME (165). This result seems to contradict the observed effects of SPMs on the increased production of such cytokines (IL-10, TGF-b) by macrophages, in a pro-resolving context, thus questioning about a potential immunosuppressive role of SPMs when modulating immune related activities in TAMs. However, these diverging effects were not observed in the same context, *i.e.* non-tumor versus tumor environment. Such results should consider the global environment, as other cytokines may also operate in regulating those mechanisms. Further studies are needed to establish a broader secretion profile of cytokines produced by macrophages upon SPM treatment in tumor context. In addition, no immunosuppressive effects have been reported in experimental models using SPMs so far, contrarily to their anti-inflammatory and pro-resolving functions. Dedicated experiments should be conducted to confirm the absence of immunosuppressive events associated with the use of SPMs in cancer context.

**Table 2:** SPMs’ effects on human macrophages in a cancer context.

Macrophages	SPMs	Effects	References
Human monocyte-derived macrophages	RvD1 – RvD2 – RvE1 (1pM – 100nM)	Increase phagocytosis of tumor cell debris and reduce pro-inflammatory cytokine secretion	(86)
Human monocyte-derived TAM	LX4 **(ATL-1)** (10nM)	Suppress TAM phenotype Decrease IL-10 secretion	(165)
Human monocyte-derived macrophages	LXA4 - RvD1 – RvD3 (AT-SPM) (100pM-100nM)	Increase phagocytosis of tumor cell debris and reduce pro-inflammatory cytokines secretion	(166)
THP-1 monocytes	RvD1-RvD2 (1-100nM)	Suppress TAM phenotype	(167)

Bold, SPM analogs or receptor agonists.

Overall, SPMs turn out to be potent modulators of TAMs’ phenotype toward a more phagocytic and less inflammatory phenotype that may boost their intrinsic antitumor activities. Altogether, these data evidenced that SPMs can directly act on macrophages and modulate their phenotype and biological activities. However, it still remains difficult to reach a consensus on a well-defined macrophage phenotype induced by SPM due to the complexity of macrophages’ polarization in *in vitro* experiments, in direct relation with their high intrinsic plasticity. Indeed, several parameters such as inter-donor variability, variety in protocols to perform *in vitro* macrophage differentiation, diversity in M1/M2 markers used for phenotype analyses by flow cytometry and/or cytokine secretion in response to LPS stimulation, all together, these discrepancies feed inter-laboratory results diversity that complicates a synthetic view of macrophage biology (70, 169). As discussed earlier in this review, there is a need for the deep characterization of pro-resolving macrophages in human context. More data about SPMs’ production by macrophages or TAMs and their effects on these cells would allow a better understanding of how SPMs modulate regulatory functions of macrophages, particularly in the context of immune networks.

### SPMs Restrict Carcinogenesis and Cancer Progression

SPMs’ anticancer effects have been evaluated in different *in vitro* and *in vivo* cancer models during either carcinogenesis or tumor progression, and are listed and referenced by SPMs and type of cancer in **Table 3**. First, SPMs can prevent inflammation-induced carcinogenesis due to their pro-resolving properties. For instance, LXA4 suppressed early development of colorectal cancer and cancer transformation in skin papillomas (38, 174) and MaR1 prevented cancer transformation after UVB-long exposure (181). Secondly, all SPM subfamilies were shown to reduce tumor growth in different murine tumor models as exposed in **Table 3**. As such, LXA4 was efficient in reducing colorectal, hepatocellular, melanoma, lung and breast carcinoma tumor growth *in vivo (* 38, 165, 166, 170, 171, 180). Resolvins of series D and E also exhibited antitumor activities on primary tumor growth in lung, lymphoma, melanoma, pancreatic and prostate cancer (86, 180). In oral squamous cell carcinoma, RvD2 showed *in vitro* and *in vivo* dose-dependent antitumor effects but RvD1 appeared less efficient to induce tumor reduction, possibly underlining cancer or specific SPMs effects (179). LXA4 and D-series resolvins also exhibited anti-metastatic effects in various murine tumor models including lung, liver and pancreatic cancers (86, 172, 176, 180).

**Table 3:** List of publications reporting SPMs’ antitumor activity in murine cancer models.

SPMs	Cancer Models	Molecules	Effects	References
LXA4	Colorectal cancer	Native	Suppress early development of colorectal cancer (Mϕ)	(38)
		**BML-111 or AT-LXA4**	Inhibit tumor growth	(166, 170)
	Hepatocellular carcinoma	**BML-111**	Inhibit tumor growth	(170, 171)
		**BML-111**	Inhibit EMT and metastasis (Mϕ)	(172)
		**BML-111**	Inhibit proliferation, invasion and angiogenesis of cancer cells (Mϕ)	(173)
	Papillomas	Native	Inflammation resolution Reduce the risk of cancer transformation	(174)
	Melanoma	**ATL-1, BML-111**	Inhibit tumor growth Alter TAM phenotype *in vivo* (Mϕ)	(165, 170)
		**ATL-1**	Decrease monocyte infiltration in tumor (Mϕ) Alters TAM phenotype *in vivo* (Mϕ)	(175)
	Pancreatic	**BML-111**	Reduce liver metastases	(176)
RvD1	Hepatocellular carcinoma	Native	Block CAF pro-tumor effects on tumor growth and metastases	(177)
		Native	Prevent liver injury and cancer transformation	(178)
	Melanoma	Native	Reduce pulmonary metastasis	(86)
RvD2	OSCC	Native	Tumor growth reduction (Mϕ)	(179)
	Lung, Melanoma	Native	Reduce pulmonary metastasis	(86)
RvE1	Hepatocellular carcinoma	Native	Prevent liver injury and cancer transformation	(178)
RvD2, RvD3, RvD4	Lung, Lymphoma, Melanoma	Native	Reduce metastases and improve survival (preoperative context)	(180)
RvD1, RvD2, RvE1	Lung, Pancreatic cancer, Prostate	Native	Inhibit tumor growth	(86)
RvD1, RvD3	Lung	**AT-RvD1, AT-RvD3**	Inhibit tumor growth	(176)
MaR1	Skin inflammation	Native	Prevent cancer risk following UVB irradiation	(181)

Bold, SPM analogs or receptor agonists.

Please refer to Prevete et al. (182) for previous studies reported SPMs’ anticancer effects (182). Mϕ indicated studies where SPM anticancer effects were mediated through counter regulation of TAM protumor functions or TAM repolarization.

Importantly, these studies showed that SPMs mediate their antitumor actions in part by counteracting pro-tumorigenic properties of TAMs, including stimulation of tumor cell proliferation and migration, epithelial-mesenchymal transition (EMT), angiogenesis and production of tumor growth-supportive cytokines and chemokines (38, 165, 167, 172, 173). As mentioned above, SPMs altered TAMs’ phenotype both *in vitro* and *in vivo (* 165, 175) and stimulated apoptotic tumor cell clearance (179).

In addition, SPMs can directly modulate various cancer cell autonomous mechanisms, including proliferation, cell death decision or invasive phenotype, as previously reported (176–178, 182). Some effects were mediated by down-regulation of the NF-κB pathway (178), also involved in cancer progression (183) and transcription of tumor-promoting genes in TAMs (164). However, no direct evidence of SPMs’ effects on NF-кB were reported on TAMs so far. Of note, beyond acting on macrophages, SPMs can also modulate other immune cells present in TME such as T- or B-lymphocytes (170), cancer-associated fibroblasts (CAFs) (177) or tumor-associated neutrophils (TANs) (184) to unlock their antitumoral activities.

**Figure 4:**
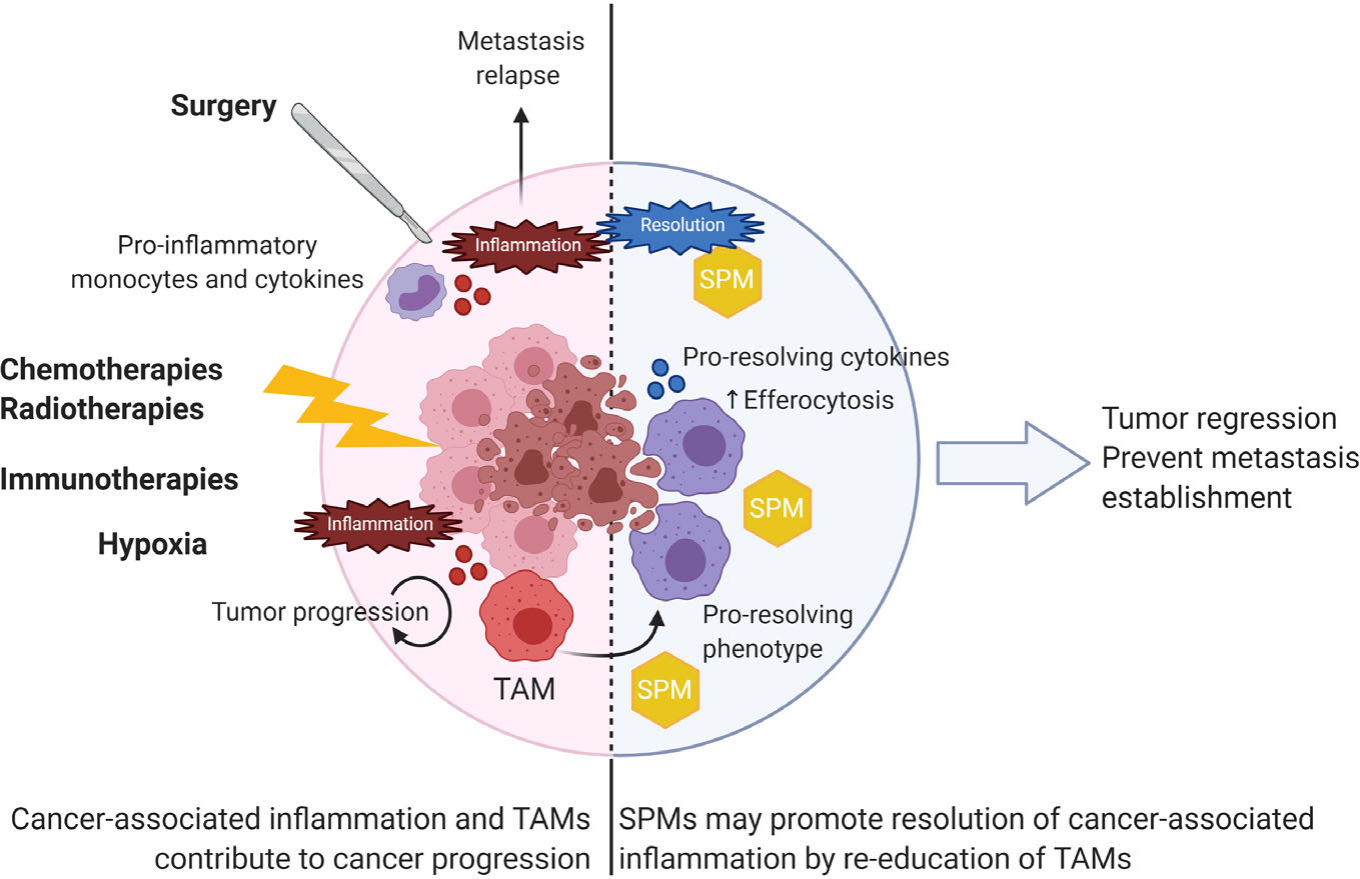
SPMs as anticancer agents to resolve cancer-associated inflammation. Various extrinsic factors such as surgery and anticancer treatments as well as intrinsic hypoxia contribute to local inflammation within tumors. TAMs largely contribute to fuel tumor progression by various functions described in **Figure 3**. Use of SPM would resolve cancer-associated inflammation by repolarizing TAMs toward a pro-resolving phenotype with increased efferocytosis capabilities. As a result, SPMs could induce tumor regression and prevent further metastasis establishment.

Altogether, these data suggest that SPMs exhibit potent anticancer activities through both direct effects on tumor cells and/or through the modulation of TAMs toward a less immunosuppressive and antitumoral phenotype (**Figure 4**). Since macrophages represent the major component of immune cells in many tumors and are key actors in SPMs’ biology, developing new therapeutic strategies based on SPM-induced stimulation of TAMs pro-resolving functions could be envisioned as a new perspective in cancer treatment.

## How to Use SPMs in Cancer Treatment?

### SPMs as Anticancer Molecules

As mentioned above, counteracting inflammation has been viewed as a therapeutic opportunity in cancer treatment for many years. NSAID and among them aspirin have already achieved encouraging results in clinical trials, showing protective effects in many cancers (42, 43). Nevertheless, the lack of specificity of NSAID gave rise to significant side effects (bleedings, cardiovascular and kidney toxicity) and infectious complications, therefore compromising their use in cancer patients (86, 89, 140). In contrast, SPMs have not shown immunosuppressive effects in experimental models of pain and inflammation (185) or cancer (86) so far. Thus, SPMs would reduce intrinsic cancer-associated inflammation and reinforce antitumor immune responses while avoiding NSAID deleterious side effects.

Surgery is often the first approach for therapeutic care practiced in several cancers such as breast and colorectal cancer (186, 187). However, several studies highlighted the risk of early metastatic occurrence after surgery by outgrowth stimulation of dormant metastasis. The surgical procedure is not immune silent as it triggered local and systemic inflammatory reactions as evidenced by increased inflammatory circulating monocytes and pro-inflammatory cytokines (188). Preventing inflammatory events and stimulating inflammation resolution by preoperative administration of RvD2, RvD3 and RvD4 inhibited the development of micro-metastases in several murine resection tumor models (180). More generally, resolvins have been shown to inhibit metastasis in cancer murine models with higher efficacy when combined to chemotherapy (**Table 3**) **(** 86). Thus, SPMs could be used in oncology as a preoperative strategy to resolve surgery related-inflammation and to prevent metastases relapse.

Importantly, anticancer treatments (chemo- and radio-therapies or immunotherapies) induce direct or indirect tumor cell death resulting in the generation of apoptotic tumor cell debris (86). Those debris largely contribute to fuel local inflammation by activating macrophages that further support cancer progression. Breaking down this vicious circle by resolving tumor-associated inflammation would stop further inflammatory and pro-tumorigenic signals. From this perspective, Sulciner and colleagues have studied the anticancer effects of resolvins in murine models of tumor cell debris-stimulated tumor growth (86). RvD1, RvD2 and RvE1 inhibited tumor growth at a higher extent than conventional chemotherapies or anti-inflammatory drugs. Moreover, synergistic antitumoral effects were observed when mice were treated with chemotherapies (gemcitabine or cisplatin) or relevant targeted anticancer therapies (cetuximab or erlotinib) in combination with resolvins, in both debris-stimulated tumor models, or spontaneous cancer models (86). Therefore, resolvins could be added as a complement to anticancer treatments to enhance therapeutic efficacy. Interestingly, compared to inflammatory drugs, resolvins have not shown immunosuppressive effects yet and were able to stimulate macrophages’ efferocytosis. Thus, resolvins represent a great alternative to the use of anti-inflammatory drugs to stimulate TAM dependent pro-resolving mechanisms during anticancer treatments without apparent immunosuppressive side effects.

Hence, several possible approaches could be envisioned to include SPM in cancer care: (1) SPM could replace anti-inflammatory drugs in perioperative surgery to prevent further inflammatory events; (2) SPM could be administered combined with chemo- and radio-therapy or immunotherapies to resolve resulting inflammation and dampen anticancer treatment side effects; (3) SPM could be envisioned in metastatic cancer treatment to prevent further metastatic relapse. Further studies are needed to evaluate the feasibility and efficacy of such approaches in human clinical studies.

### Engineering SPM Mimetics

One drawback of SPM is their short lifespan due to their lipidic nature and rapid metabolic inactivation, which can make them difficult to use. For instance, LXA4 added to macrophages results in LXA4 loss within minutes of its exposure (189). There has been an impetus for the development of small molecules or analogs capable of mimicking their biological activities but with a greater stability (**Tables 4**, **5**).

**Table 4:** SPMs or AT-SPM analogs and receptor agonists.

Nature	SPMs	Name	Reference
Analogs	LXA4	15(R/S)-methyl-LXA4, 16-phenoxy-LXA4	(190)
	PD-1	22-F-PD1	(162)
	MaR1	7S-MaR1	(191)
	RvE1	19-(*p*-fluorophenoxy)-RvE1 methyl ester	(27)
AT-SPM analogs	LXA4	ATL-1 (15-epi-16-(para-fluoro)phenoxy-LXA4)	(152, 153, 165, 175)
Receptor agonists	FPR2/LXA4	BML-111 (5(S),6^®^,7- trihydroxyheptanoic acid methyl ester)	(170–173)
	GPR32	NCGC00120943 (C1A), NCGC00135472 (C2A) pMPPF, and pMPPI	(19)
	ChemR23	Monoclonal antibody	(192)

Here are listed SPM mimetics, including analogs, AT-SPM analogs and receptor agonists that show increased stability and biological activities in inflammation resolution settings.

**Table 5:** Therapeutic approaches for the development and use of SPM mimetics in cancer treatments.

			Disadvantages	Advantages
**SPMs**	Native		Short half-life	Biological activities
	Analogs		Short half-life	Increased Stability
				Biological activities
	Receptor agonists	Small molecules	Short half-life	Increased Stability
				Easy to develop
				Biological activities
		Monoclonal antibodies	Manufacturing costs	Long exposure for chronic disease
				Easy to develop
				Specificity and selectivity

#### SPM Analogs: Chemical Modifications of Native SPMs

In the late 1990’s, Serhan and colleagues designed the first LXA4 analogs with chemical modifications of native LXA4, resulting in increased stability due to enzyme conversion resistance (189). AT-SPM generated by low-dose aspirin also present increased stability compared to native SPM (44). Since several SPM analogs characterized by a higher stability both *in vitro* and *in vivo* have been developed (27, 189). Importantly, these analogs demonstrated similar biological activities than native SPMs *in vitro* and *in vivo.* They were able to stimulate macrophages pro-resolving functions and induce inflammation resolution in a GPCR-dependent manner in *in vitro* and *in vivo* inflammatory models (19, 162, 191). These analogs also exerted antitumor effects, including inhibition of primary tumor growth and metastasis as previously detailed in **Tables 2** and **3** **(** 165, 170, 175, 193). Of note, analogs displayed biological activities at doses lower than native SPMs (189). Specifically, AT-RvD1, AT-RvD3 and AT-LXA4 have been shown to inhibit tumor growth in colorectal, lung, and breast murine models at doses 1,000-fold lower than aspirin (166).

#### SPM Receptor Agonists: Targeting SPM Specific Receptors

Among identified SPM receptors to date, ChemR23 (RvE1), ALX/FPR2 (LXA4, RvD1), GPR32 (LXA4, RvD1, RvD3), GPR18 (RvD2) and LGR6 (MaR1) are GPCRs that are often expressed on macrophages’ surface (19, 194). Development of specific agonists for SPM receptors would mimic SPMs’ activities on macrophages and could be used to modulate their biological activities. Of note, GPCRs are currently the most important group of targets for approved drugs (195).

As SPM and their corresponding binding pockets in GPCRs are small, it is thus quite easy to develop specific receptor agonists (28) (**Tables 4** and **5**). This process requires a high understanding of the targeted receptors’ pharmacology as underlined by functional selectivity for one ligand to certain downstream signaling pathways (referred as ligand bias theory) (28). Drug discovery and challenges regarding GPCRs in relation to inflammation resolution have been extensively reviewed here (15).

Among receptor agonists, BML-111 is a commercially available synthetic molecule that targets the LXA4 and RvD1 receptor ALX/FPR2 (196). BML-111 has shown antitumor activities in murine colorectal, hepatocellular and melanoma tumor models (170) (**Table 3**).

Therapeutic antibodies can also be used as agonists to activate GPCRs (192). They offer several advantages: longer life span than small molecules, specificity and selectivity (197). In a recent report, Trilleaud et al. illustrated that a selected and optimized agonist monoclonal antibody can exercise pro-resolving RvE1 actions on pro-resolving macrophages’ polarization, neutrophils migration inhibition and acceleration of neutrophils’ apoptosis. While this pro-resolving agonist antibody accelerates inflammation resolution *in vivo* in various models, and was also able to trigger efficient resolution in non-resolving chronic inflammatory models, Trilleaud et al. also reported that such pro-resolving agonist antibody can significantly limits colon carcinogenesis induced by inflammation and eradicate established colorectal tumors in some mice in monotherapy or combination with chemotherapy (192). Developing specific and lasting agonists may be a promising approach for the modulation of both inflammation resolution and antitumor responses in chronic diseases such as cancers.

### SPM Mimetics in Clinic

As SPM and their mimetics have shown encouraging results in preclinical models, these molecules have been further tested in human and shown both safety and efficacy (**Table 6**). SPMs are immunoresolvent rather than immunosuppressive molecules that exert their pro-resolving and anti-inflammatory actions at low doses without deleterious side effects, which makes them very interesting molecules on the clinical level (201). Those results in human are encouraging to pursue the development of small molecules capable of mimicking SPM to stimulate pro-resolving functions of macrophages/TAM.

**Table 6:** SPMs and SPM mimetics tested in human.

SPMs	Analog/Receptor agonist	Clinical trials	Effects	References
RvE1	RX-10045 **(analog)**	Combined Phase I and II in patients with dry eye symptoms	Safe and well tolerated	NCT00799552
LXA4	15(R/S)‐methyl‐lipoxin A_4_ **(analog)**	Study in Infantine eczema	Well tolerated Reduction of eczema severity	(198)
LXA4	BLXA4-ME **(analog)**	Phase I/II in patients with gingivitis (on going)	Assess safety and preliminary efficacy	NCT02342691
LXA4	LXA4 **(native)**	Study in patients with asthma	LXA_4_ attenuated leukotriene C4-induced bronchoconstriction	(199)
LXA4	5(S),6(R)-LXA_4_ methyl ester **(analog)**, BML-111 **(agonist)**	Pilot study in asthmatic children	Safe and well tolerated Improvement of pulmonary functions	(200)

## Conclusion

Cancer and inflammation are closely related not only during initial steps of carcinogenesis but also during cancer progression as well as tumor response to anticancer treatment including modern immunotherapies. Hijacked macrophages within tumor microenvironment largely support tumor growth in part by sustaining local immunosuppression. Due to the emerging central role of SPMs in inflammation resolution, SPM biology is now a trending field of study in human diseases that emerge as potent therapeutic targets not only in chronic inflammatory diseases but also in cancers. Due to macrophages’ high plasticity, SPMs may indeed represent a new approach for cancer treatment by repolarizing TAMs toward antitumor macrophages with the advantage of using bioactive molecules, supposedly without immunosuppressive properties and deleterious side-effects. Developing stable and lasting SPM, these data confirm the value of targeting SPMs by promoting pro-resolving responses in a context of cancer treatment.

## Author Contributions

ML performed the scientific literature search, design the figures and wrote the review. VG and NP completed the review. SB-N and CB completed and finalized the review. All authors contributed to the article and approved the submitted version.

## Conflict of Interest

ML, VG, and NP are employees and/or shareholders of OSE Immunotherapeutics, a company developing pro-resolutive agonist monoclonal antibodies. VG and NP are patent owners on ChemR23 agonist monoclonal antibodies.

The remaining authors declare that the research was conducted in the absence of any commercial or financial relationships that could be construed as a potential conflict of interest.

## Abbreviations

AA, Arachidonic acid; APCs, Antigen-presenting cells; AT-SPM, Aspirin-triggered SPM; cAMP, Cyclic adenosine monophosphate; CAFs, Cancer-associated fibroblasts; CAR, Chimeric antigen receptor; COXs, Cyclooxygenases; CSF-1, Colony-stimulating factor 1; DHA, Docosahexaenoic acid; EGF, Epidermal growth factor; EPA, Eicosapentaenoic acid; GM-CSF, Granulocyte-macrophage colony-stimulating factor; GPCRs, G protein-coupled receptors; IFN, Interferon; IL, Interleukin; iNOS, Inducible nitric oxide synthase; LOXs, Lipoxygenases; LPS, Lipopolysaccharides; LTs, Leukotrienes; LTB4, Leukotriene B4; LXs, Lipoxins; MaR, Maresin; MDSC, Myeloid-derived suppressor cells; MHC, Major histocompatibility complex; NF-kB, nuclear factor-kappa B; NK, Natural Killer; NO, Nitric oxide; NSAID, Nonsteroidal anti-inflammatory drugs; PGs, Prostaglandins; PGE2, Prostaglandin E2; PMNs, Polymorphonuclear neutrophils; PUFA, Polyunsaturated fatty acids; ROS, Reactive oxygen species; Rv, Resolvins; SPMs, Specialized pro-resolving mediators; TAMs, Tumor-associated macrophages; TANs, Tumor-associated neutrophils; TGF-β, Transforming growth factor beta; TLR, Toll-like receptor; TME, Tumor microenvironment; TNF-α, Tumor Necrosis Factor; VEGF, Vascular Endothelial Growth Factor.
